# Engaging and supporting women with chronic kidney disease with pre‐conception decision‐making (including their experiences during COVID 19): A mixed‐methods study protocol

**DOI:** 10.1111/jan.14803

**Published:** 2021-03-03

**Authors:** Rhiannon Phillips, Leah McLaughlin, Denitza Williams, Helen Williams, Jane Noyes, Caron Jones, Catherine Oleary, Carmen Mallett, Sian Griffin

**Affiliations:** ^1^ Cardiff School of Sport and Health Sciences Cardiff Metropolitan University Cardiff UK; ^2^ School of Health Sciences Bangor University Bangor UK; ^3^ Patient Representative Cardiff UK; ^4^ Betsi Cadwaladr University Health Board North Wales UK; ^5^ Department of Nephrology and Transplant Cardiff & Vale University Health Board Cardiff UK; ^6^ Swansea Bay University Health Board Swansea UK

**Keywords:** kidney disease, mixed‐methods, nursing, pregnancy, protocol, shared decision‐making, women

## Abstract

**Aim:**

To report a protocol for a qualitative study to better understand the key factors that influence decision making about pregnancy from women's perspectives and to use these data to develop a theoretical model for shared decision‐making tools for the multiple stakeholders.

**Design:**

Mixed‐method design using online surveys (with validated components) and purposively sampled follow‐up semi structured interviews.

**Methods:**

Funded from September 2020 for 12 months. Online surveys of adult women (aged 18–50) identified via all Wales kidney database (n ≥ 500), additional recruitment through multidisciplinary healthcare professionals, relevant third sector organizations and social media. Follow‐up in‐depth qualitative interviews with n = 30 women. Linear regression models to identify associations between shared decision‐making preferences and clinical and psychosocial variables. Qualitative interviews will use a visual timeline task to empower women in taking control over their narratives. Qualitative data will be fully transcribed and analysed thematically, based around a chronological and theoretical (theoretical domains framework) structure that maps out key challenges and opportunities for improved decision support in the care pathway. Visual timelines will be used during stakeholder consultation activities, to enable us to co‐create a map of current support, gaps in provision, and opportunities for interventions. Quantitative data will be analysed descriptively to characterize our cohort. We will assemble a multidisciplinary shared decision‐making intervention development group and provide ongoing stakeholder consultation activities with patient and public representatives.

**Discussion:**

Outcomes will support new learning into; the ways women's knowledge of kidney disease may affect family planning and pregnancy, their needs in terms of psychological and social support, and how they weigh up the pros and cons of starting a family.

**Impact:**

Evidence will inform the design of new shared decision‐making tools to better support women with the complex and often emotional decisions about having children while living with kidney disease.

## 1 INTRODUCTION

An estimated 195 million women are affected by kidney disease worldwide. (‘^2018^ WKD Theme ‐ World Kidney Day’, n.d.) Recent research increasingly highlights that many aspects of kidney disease have specific gender disparities (Bikbov et al., 2018; Goldberg & IIan, n.d.; Iseki, 2008; Nature Publishing Group, 2018; Piccoli et al., 2018). Differences in global healthcare systems, culture, socio economics, attitudes and values have led to a global call to action for new research which puts these contradictions into their respective contexts (Bikbov et al., 2018). Decisions about pregnancy and the challenges pregnancy can bring while living with kidney disease is one such difference and unique to women. Clinical nurse specialists from nephrology, transplantation, gynaecology, obstetrics and midwifery work with multidisciplinary teams (MDTs) to provide care, information and support to women with kidney disease who are considering pregnancy. Recent research in developed countries has focussed on learning more about experiences of care and support from the women's perspectives and found among other things, an unhelpful focus on clinical perspectives of risk, and importantly a need for new shared decision‐making interventions to support patients and professionals with the complex and often emotional decisions which need to be made about pregnancy while living with kidney disease (Jesudason & Tong, 2019; Tong et al., 2015).

## 2 BACKGROUND

The presence of maternal chronic kidney disease (CKD) is well‐recognized to be associated with adverse pregnancy outcomes, and medical management of these women during the course of their pregnancy can be challenging. The risks to both the mother and baby increase steeply with declining kidney function, in particular for those with CKD stage 4 or 5, on dialysis or with a kidney transplant.

Globally more than 3 million people are currently receiving renal replacement therapy (RRT), with this projected to rise to 5.5 million by 2030 (Renal Registry UK, 2015). More women have kidney disease than men, yet more men are on a renal replacement therapy. Gender disparities such as this are becoming increasingly apparent within the global kidney population (Antlanger et al., 2019). Approximately 5% of Australian women of childbearing age have albuminuria or abnormal eGFR (AIHW, 2014). In England, an estimated 2.5% of women in the 35–54 age group have CKD stage 3–5, putting them at increased risk of pregnancy‐related complications such as pre‐eclampsia (Public Health England, 2014). Wales is part of the United Kingdom with a devolved healthcare system and a population of around 3 million (Wales Population Database, 2020). The prevalence of CKD rises with age and the number of births to mothers aged 35 and over in Wales is also steadily increasing (Welsh Government, n.d.). The need to engage and support women of reproductive age with CKD with decisions about pregnancy and family planning is a necessary cornerstone of providing high‐quality patient‐centred care in CKD (Jesudason & Tong, 2019).

Decisions about pregnancy are complex and emotive, and for women with CKD, pregnancies need to be carefully planned and monitored. Some medications used by CKD patients can be teratogenic and may need to be stopped or changed well in advance of pregnancy. There is a significant risk that pregnancy will lead to an irreversible decline in kidney function, a risk that increases with the baseline severity of disease (Blom et al., 2017). The risk of preterm delivery is significantly greater for women with CKD 4 and 5 (89% of births at <37 weeks) and is often accompanied by intra‐uterine growth retardation.

Qualitative studies indicate that effects of pregnancy on long‐term renal health, and of CKD on pregnancy outcomes, are major concerns for women with CKD (Tong et al., 2015a, 2015b). Women report ‘decisional conflict’ surrounding family planning and express a need for ‘control and determination’ over their choices (Tong et al., 2015b). Patient‐health professional relationships can affect physical health outcomes for women who give birth after a renal transplant, as this forms an important component of the social support that women receive (Yoshikawa et al., 2018).

Shared decision making is an approach where clinicians and patients share the best available evidence and patients are supported to consider their options and preferences (Elwyn et al., 2012). Shared decision making can help patients make more informed decisions aligned with their personal preferences, become more active and empowered in their own healthcare, have better relationships with their healthcare professionals and feel more satisfied with their choices (Foundation, 2012). Preconception decision aids for women with rheumatoid arthritis, multiple sclerosis (Prunty et al., 2008) and epilepsy (McGrath et al., 2017) have been found to increase women's knowledge about pregnancy and their disease and reduce decisional uncertainty.

### 2.1 Rationale

There are significant gaps in the evidence on the childbearing‐related information needs of women with non‐communicable diseases (Holton et al., 2012) and the effectiveness of interventions to improve outcomes for women with disabilities who are starting a family and their children (Malouf et al., 2014). Qualitative research on shared decision making has been identified as a high priority area in the latest Renal Association Clinical Practice Guideline on Pregnancy and Renal Disease (Wiles et al., 2019).

This study will address an important gap in the evidence base by clarifying how women of reproductive age who have CKD make decisions about pregnancy, and how this relates to their health, well‐being and pregnancy outcomes. These data will form part of a systematic process of developing a preconception shared decision‐making intervention to facilitate informed and preference‐sensitive discussions between clinicians, specialist nurses, women with CKD and their families about reproductive choices.

Implementing shared decision making during the pre‐conception period with women who have CKD has the potential to improve the quality of decisions made about their care, helping avoid potential disadvantages to many women with CKD and their families (Elwyn et al., 2012).

### 2.2 Theoretical framework

**FIGURE 1:**
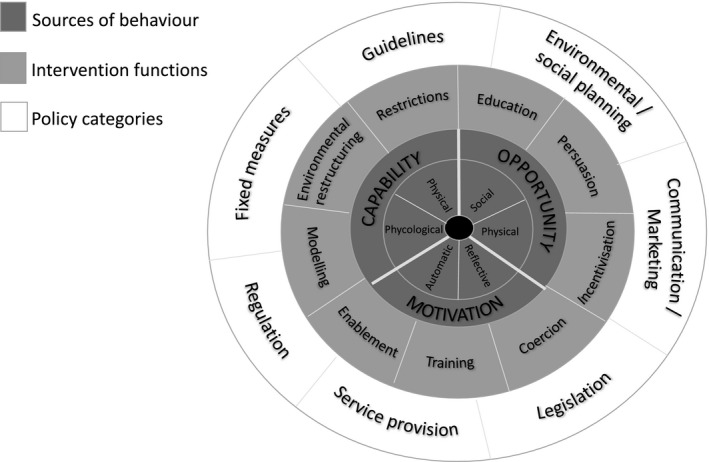
The Behaviour Change Wheel, adopted from Michie, et al. ‘The behaviour change wheel: A new method for characterizing and designing behaviour change interventions’.

**FIGURE 2:**
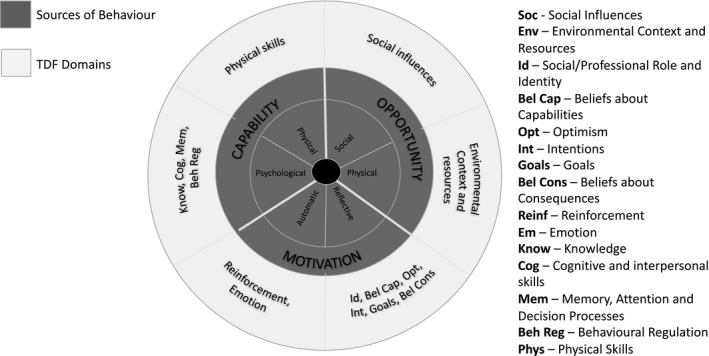
Theoretical Domains Framework cited from Cowdell, et al. ‘How is the theoretical domains framework applied to developing health behaviour interventions? A systematic search and narrative synthesis’.

This is a mixed‐methods study using surveys and depth qualitative interviews with women aged 18–50 living with CKD across the United Kingdom. We will apply a combination of the behaviour change wheel (BCW) (Figure 1) and the theoretical domains framework (TDF) (Figure 2) to support any designs and evaluations of behaviour change interventions and policies resulting from the study (Richardson et al., 2019).

Combined these frameworks will enable us to design new patient‐centred shared decision‐making interventions (including who does what, when and where), map these onto wider health and social contexts, and identify areas in health and psycho/social care where interventions are (a) needed and (b) most likely to work and to replicate them in future.

Theoretically informed behaviour change interventions are especially important in kidney health and social care as the system is very complex: large MDTs (including social workers, psychologists, dieticians, pharmacists, occupation therapists, specialist nurses, renal registrars and renal consultants) are tasked with long‐term patient care. These MDTs work closely with kidney charities and wider social service providers. Women who are making decisions about pregnancy, or are pregnant will also consult with obstetrics and gynaecology specialists. Women who have kidney failure will also need to make decisions about their future treatment (e.g., dialysis and transplant) in addition to pregnancy options and choices.

The TDF and BCW provide a comprehensive eight‐stage process to intervention design (a) define the problem, (b) select the target behaviour, (c) specify the target behaviour and identify (d) what needs to change, (e) intervention functions, (f) policy categories, (g) behaviour change techniques (BCTs) and (h) mode of delivery (Cowdell & Dyson, 2019). The framework(s) are suitably robust and flexible enough to underpin theoretical modelling in the complex system of kidney health and social care.

## 3 THE STUDY

### 3.1 Aim(s)

To improve support for women with kidney disease with the complex choices they need to make in relation to pregnancy by:

1. Identifying needs and preferences of women of reproductive age with kidney disease by improving our understanding of how women make decisions about pregnancy, and investigating associations between pregnancy, health, well‐being and psychosocial contexts
2. Constructing a theoretical model for decision making in relation to pregnancy, an essential first step in the development of a preconception shared decision‐making intervention for use in clinical practice

### 3.2 Objectives

To:

1. carry out an online survey of women of reproductive age living in the United Kingdom who have kidney disease to understand women's information needs, decision‐making preferences, priorities and psychosocial context regarding their own health, well‐being and healthy pregnancies.
2. obtain consent to follow‐up from women completing the online survey, with a view to establishing a longitudinal cohort that will enable us to prospectively investigate long‐term health and well‐being outcomes for women with kidney disease and their children in the future.
3. use in‐depth qualitative interviews to understand women's lived experiences and develop a theoretical model of how women make decisions about pregnancy using the TDF and BCW (Michie et al., 2011).
4. carry out a variety of stakeholder consultation activities to identify opportunities in the current care pathway to introduce enhanced support with decision making based on women and MDTs experience and knowledge of services already available in Wales for women with kidney disease (e.g. preconception counselling) and establish which types of decision support tools are preferred by women and MDTs (e.g. value clarification exercises, option grids).
5. Facilitate recruitment into the UK‐wide Rare Renal Registry (RaDaR, rarerenal.org) in parallel with this work to help improve the availability of high‐quality evidence to inform women and MDTs in the future.

### 3.3 Design/Methodology

**FIGURE 3:**
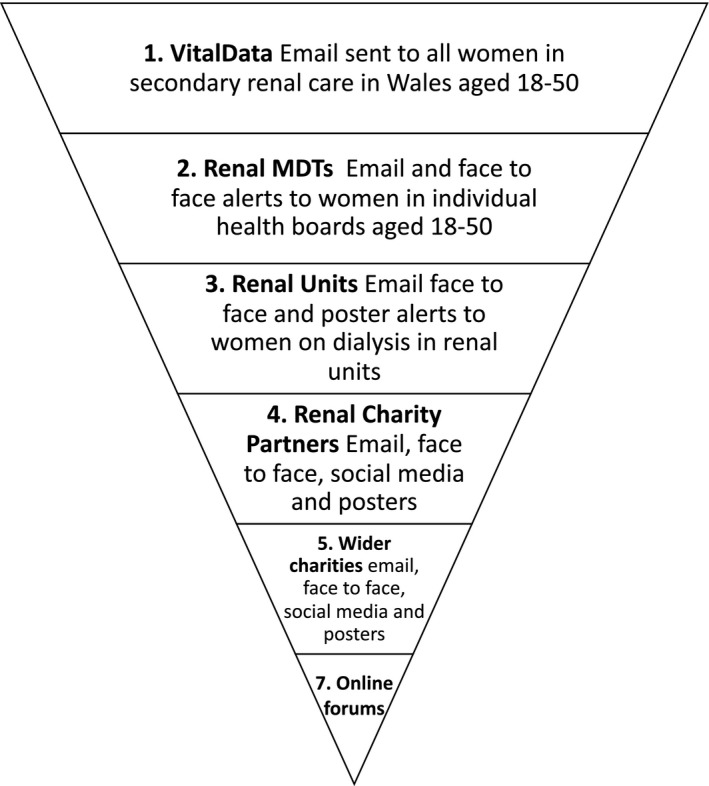
Recruitment to survey systems and services.

This is a mixed‐method study adopting a convergent design to data collection and analysis (Figure 3) (Noyes, Booth, et al., 2019). This will include an online survey of women of reproductive age using kidney services across Wales (n ≥ 500), in‐depth qualitative interviews with up to 30 women of reproductive age who have kidney disease and ongoing stakeholder consultation activities with patient and public representatives and a multidisciplinary intervention development group. Shared decision making in practice is highly complex incorporating consideration of a patient's clinical history, lifestyle, social circumstances and preferences (Elwyn et al., 2012). As such, we will follow the Medical Research Council guidance on developing and evaluating complex interventions (Craig et al., 2008; Skivington et al., 2018) and the International Patient Decision Aid Standards (IPDAS) guidelines (Coulter et al., 2013; Elwyn et al., 2009). This study will correspond to the ‘modelling’ phase of the MRC guidance and the scoping, steering and design (components 1 and 2) stages of the IPDAS guidance on developing decision aids (Coulter et al., 2013; Craig et al., 2008).

### 3.4 Pan‐Wales survey of women of reproductive age with kidney disease

The online self‐report survey (FORM D) will provide us with cross‐sectional baseline data for a population of women of reproductive age with kidney disease to enable us to understand their decision‐making processes, lived experiences, and health and well‐being outcomes relating to pregnancy. The survey will also allow us to apply a purposive sampling framework to recruit women for the in‐depth qualitative interviews, as well as establish a cohort of women of reproductive age who have kidney disease, which will provide an opportunity for prospective longitudinal data to be collected in the future.

#### 3.4.1 Participants and setting

##### Inclusion criteria

All women with kidney disease, or who are on dialysis, or have a transplant aged between 18–50 and living in the United Kingdom will be eligible to participate in this study. As a Wales led study the primary focus and settings will be in Wales, and at this stage, focus on the Welsh perspective. In Wales approximately 5000 women aged 18–50 are in secondary kidney care and will attend clinics at one of the following three health boards; Swansea Bay, Betsi Cadwaladr and Cardiff and Vale which cover five sites across Wales (Cardiff, Swansea, Wrexham, Bangor and Glan Clwyd).

##### Exclusion criteria

‐ Women under 18 and over 51.
‐ Women who live outside the United Kingdom
‐ Men

##### Measures

The survey (FORM D) will include questions on current treatment for kidney disease (drop down list); renal function (eGFR); years since diagnosis of kidney disease; current medication (drop down list); co‐morbidities (open text) and family situation (currently pregnant, planning to try to get pregnant, and/or had a pregnancy, had children already, and if so, how many and what their ages are). Demographic data will be collected on age, highest educational qualification, geographical location (postcode), marital/relationship status, ethnicity and current employment status. The survey will also assess information needs in relation to pregnancy and family planning, preferences for shared decision making relating to pregnancy (Autonomy Preference Index), and open questions relating to current experiences of shared decision making, gaps in provision and preferences for decision support.

##### Procedure

**FIGURE 4:**
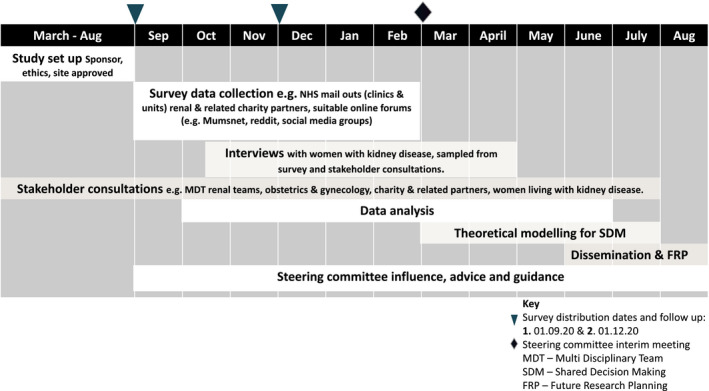
Study flow chart and diagram. [Colour figure can be viewed at wileyonlinelibrary.com]

The self‐report survey will be completed online using the Bristol Online Survey system. Recruitment for the online survey will take place using a multi‐pronged approach (Figure 4) including publicising the survey pan‐Wales via nephrology clinics, social media (Twitter and Facebook), and patient‐facing organizations (e.g., Kidney Research UK, Paul Popham Foundation, Kidney Wales). The informed consent process will be completed online prior to commencing the survey (FORM C) and include an email cover letter (FORM A) and Participant Information Sheet (PIS) (FORM B). Women who have taken part in the survey will be asked for consent to contact in the future to invite them to take part in qualitative interviews and follow‐up surveys. We will also signpost to the Registry of Rare Kidney Diseases (RaDaR). All patient facing materials will be produced bilingually.

##### Recruitment and identification

Figure 3 provides an overview of the systems and services we will connect with to promote and distribute the survey. The design focuses on women currently in secondary care in Wales, but is suitably flexible to include women with, for example early stage kidney disease and women in England who may be alerted via social media or word of mouth. This is important as we have found from previous research, it is common for people to reside in Wales and receive treatment in England and vice versa. A key part of this study is also to promote The National Registry of Rare Kidney Diseases (RaDaR), a UK wide database and open to eligible UK citizens.

We will recruit through:

1. VitalData, the renal database in use in all Welsh units.
2. The kidney MDTs, for example consultants, specialist nurses, psychologists, social workers and other key members of the MDTs in Wales.
3. Kidney unit staff and managers. There are currently 18 dialysis units across Wales. Units provide bespoke communications (newsletters, updates, social media groups) to individuals who dialyse in their units. We will approach unit managers and staff to share the survey invitation via email, face‐to‐face communications and posters visibly on display.
4. Renal Charity Partners. All kidney charities in the United Kingdom have been informed about this study and form part of the steering committee (see steering committee section) these include, Paul Popham Renal Fund (Wales), Kidney Wales (Wales), Kidney Care UK (UK) and Polycystic Kidney Disease Charity UK (UK). We will approach kidney charities to advertise the survey through their channels and networks, including social media and mailing lists.
5. Wider Charities. We will approach several national charities including; Diabetes Cymru, Diabetes UK, British Heart Foundation and SANDS (stillbirth and neonatal death) Charity UK to distribute an invitation for those who are eligible to take part.
6. Online forums. We will promote the study through online social forums such as Netmums, MumsNet, Reddit, Facebook and local social media groups (MDTs and charity partners will support us to identify local groups).

##### Sample size

Based on population analysis, there are approximately 5,000 women in Wales aged between 18–50 who have CKD or had a transplant. For the planned regression analysis, we require at least 120 participants. This will enable us to carry out analysis of multiple variables associated with information needs based on Green's (Green, 1991) rule of thumb of n = 104 + *m* for regression analysis assessing the effect of individual predictors on continuous outcomes, where *m* is the number of variables entered into the model. However, we also seek to characterize this population, collect longitudinal data in the future, and investigate the potential for linking data from our cohort to RaDaR for future analysis. Given the timeframe and resources available for the current study, we will aim to recruit at least 500 women into our cohort. By using VitalData and publicly available data via the Renal Registry, we will be able to assess an approximated response rate and describe any key differences in disease profiles/demographics/geographical area that could have an impact on the generalizability of our sample. We plan to continue building this cohort beyond the scope of the current study. Due to the use of the online survey methodology, there is potential to extend this work to target under‐represented groups identified during our initial analysis, and to open the survey out across the United Kingdom and internationally in future phases of this research at little additional cost.

##### Survey data analysis

Analysis of the quantitative data will be carried out using SPSS.v.27. Analysis will be primarily descriptive, providing an overview of the clinical and demographic characteristics of our sample, and their well‐being, shared decision‐making preferences and information needs. To identify differences in self‐reported pregnancy and health outcomes according to key clinical and demographic factors, independent t‐tests will be carried out, and 95% confidence intervals will be calculated. Between‐group differences in support received/desired, which will be coded as binary categorical variables, will be tested for using chi‐square. The Holm–Bonferroni correction (Sture, 1979) for multiple comparisons will be used to adjust the alpha level to allow for multiple comparisons. Multivariable regression analysis will be used to identify variables that are independently associated with levels of information needs and shared decision‐making preferences to identify groups that may be in need of additional or tailored support. Open text data from the survey will be coded thematically using an inductive approach to identify frequent, dominant and significant themes that emerged from the data (Thomas, 2006).

#### 3.4.2 Qualitative interviews with women with CKD

Kidney disease can impact on fertility and pregnancy outcomes and may alter the planned timing of a pregnancy. For example, women with declining function or on dialysis may elect to postpone pregnancy until they have received a kidney transplant. The experience of organ failure or organ donation can in itself change women's attitude to pregnancy, for example due to feelings of fear, guilt or shifting priorities. These aspects are unique to kidney disease and women's attitudes to these are poorly understood in the UK population and will be explored through qualitative interviews. Their need for decision support tools will be explored.

##### Participants and sampling

We will interview up to 30 women with kidney, about their lived experiences and shared decision‐making preferences, purposively sampled from survey participants who have provided consent to contact. The maximum variation sampling framework will ensure that a range of views are represented, including from those with ‘average’, ‘below average’ or ‘above average’ kidney disease burden based on the survey scores, and those who have and have not already had a pregnancy/pregnancies. The qualitative sample size and approach to sampling has been determined on the basis of providing sufficient information power to answer the research questions for this component of the study (Malterud et al., 2016).

##### Interview procedure

Informed consent (FORM E, consent for interview) will be sought before interviews take place. The qualitative interviews will be facilitated using a visual timeline (FORM F, Interview guide and timeline) (Goldenberg et al., 2016). The use of participatory approaches such as timelines in qualitative research can empower participants by allowing them to navigate the conversation, increase their level of comfort in discussing sensitive topics, provide positive moments and opportunities for closure (Goldenberg et al., 2016; Kolar et al., 2015). Members of our team have successfully used this method in both face‐to‐face and telephone interviews with women with autoimmune rheumatic diseases to discuss pregnancy planning, pregnancy and early parenting, eliciting rich narratives (Phillips et al., 2018; Williams et al., 2019).

Interviews will be carried out in a COVID safe way (see COVID update below). Before the interviews, women will be sent a resource pack, which will include stationary, a timeline template, and examples of the themes that we are interested in covering during the interview (FORM F, interview guide and timeline). Timelines will provide a visual tool to enable women to map out their journey towards starting a family, noting key events and their physical and emotional responses to these.

##### Qualitative data analysis

Interviews will be audio‐recorded and transcribed verbatim. Data will be analysed thematically using a hybrid approach of inductive and deductive analysis based on social phenomenology (Fereday & Muir‐Cochrane, 2006). NVivo will be used to facilitate analysis. The coding framework will be built around the TDF (Michie et al., 2011) (Figure 2

) to enable us to develop a theoretical model of decision‐making relating to pregnancy and provide a strong conceptual foundation for the design of a shared decision‐making intervention. We will use regular qualitative research team meetings to discuss data production, development of the coding framework and data analysis, with each member of the qualitative research group adding their unique perspective to the analysis (Barbour, 2001).

### 3.5 Overarching convergent data integration

We will use a matrix approach to data integration (O’Cathain et al., 2010) in order to look in depth at individual cases across different types of data (survey and interviews), the relationships within and between datasets, and to identify the key patterns and tendencies across all of the data. This will enable us to create a better understanding of women's lived experiences and at the same time develop the overall theoretical model.

### 3.6 Timescale and milestones

We will complete this study within 12 months. Our timescale for the study is set out below and summarized in Figure 4. ‘Study flow chart and diagram’.

Months −6 to 0: Study set up

1. Obtain favourable approvals before the study commences
2. Contracting and staff recruitment
3. Establish a stakeholder reference group

Month 1–6: Online survey and qualitative data collection.

Months 3 to 10: Qualitative survey and interview data coding and analysis.

Months 2 to 8: Quantitative survey data analysis.

Months 6–12: Development of the theoretical model for shared decision making in this context.

Months 9–12. Write‐up, dissemination of findings, and planning for future research (Figure 4).

### 3.7 Ethical considerations

We have received Research Ethics Committee approval (Registered REC Number: 20/WA/0157) Informed written consent is required for participants to take part. We attach the following participant information documents: FORM A Cover Letter; FORM B participant information sheet; FORM C Consent form survey; FORM D the online survey; FORM E consent form interview; FORM F Interview topic guide and timeline; and FORM G Poster for recruitment, as online supplemental files (Data S1).

#### 3.7.1 Specific ethical considerations for this study

This is a low‐risk study undertaking surveys and interviews with adults. Nonetheless some of the topics covered are sensitive, for example complicated pregnancies, miscarriages, stillbirths and bereavement. We have produced an ‘Ethical Considerations, Practical strategies and Distress Protocol (Data S2). This is a modified protocol which we have successfully used in previous studies with acutely bereaved participants (Noyes, Mclaughlin, et al., 2019).

We have also partnered with Stillbirths And Neonatal Death Charity (SANDS) and provide links and contact details to this charity, as well as kidney disease charities, and relationship counselling charities (Relate) at the end of the survey. Research officers conducting this study have long term experience undertaking research in these areas and are trained to be mindful of the appropriate times to pause or stop collecting data. Interviewers will sign post to the additional support charities and services at the end of each interview, in case participants would like to access free support and counselling outside of a NHS context.

### 3.8 Validity, reliability and rigour

The study has been awarded funding via a competitive funding stream (British Renal Society and Kidney Care UK research funding call) and subject to high‐quality, independent expert peer review. Validated tools have been adopted for use in the survey (Decision self‐efficacy scale, CollaboRATE, Autonomy Preference Scale) (Elwyn et al., 2013; Morandi et al., 2017; Ottawa Hospital, 2018). We will use the four‐dimension criteria (credibility, dependability, confirmability and transferability) in the interview processes and subsequent analysis (Forero et al., 2018). The MDT will bring their individual expertise to the interpretation of interview transcripts and agree key findings and messages. The results‐based convergent design will ensure all outcomes are integrated and synthesized to address the overall study aims (Noyes, Booth, et al., 2019). Reporting will follow agreed international standards to ensure transparency from the EQUATOR network (Network, n.d.).

#### 3.8.1 Stakeholder consultation activities

Our multidisciplinary intervention development group includes experts in nephrology, specialist nursing, obstetrics, clinical and health psychology, shared decision making, and development and evaluation of complex interventions. Through our professional networks, we will carry out a variety of stakeholder consultation activities, including one‐to‐one meetings and visits to MDTs and patient groups to exchange knowledge.

#### 3.8.2 Steering committee

The core research team includes MDT of health psychologists, social workers, nephrologists, health and social care scientists, and patient representatives. We have recruited additional representatives from the following: consultant gynecologists; patient representatives; shared decision‐making experts; CEO of the Paul Popham Renal Fund; lead counsellor Paul Popham Renal Fund, CEO of Kidney Wales; advocacy officer Kidney Care UK; representatives from Stillbirth and neonatal death charity (SANDs); Clinical Lecturers in Renal Sciences (Kings College London, Department Women and Children's Health) and the Welsh Renal Clinical Network (commissioners of renal services in Wales). We will continue to recruit suitable representatives to the steering committee as appropriate and will communicate with the committee throughout via emails, face‐to‐face and virtual meetings with specific groups and people. We will also host an interim steering committee meeting with the whole group. This meeting will provide independent views on progress to date and expertise and input shaping the reminder of the study—in particular the theoretical modelling—and future research planning.

#### 3.8.3 Patient and public involvement

##### Contribution to research development

The original intellectual ideas for the research emerged from consultations with women with kidney disease who as individuals, partners, mothers and carers articulated that they faced complex decision making about pregnancy and their personal health and well‐being. This grant was developed in partnership with three women living with kidney disease all of whom have faced challenges in making decisions about pregnancy. We also undertook informal consultancy with a group of eight patients who are volunteer ‘befrienders’ at the Paul Popham Foundation. All of the group felt that the research addressed an important gap in understanding women's needs, values and expectations in terms of pre‐ and post‐natal psychosocial care and support.

##### Involvement and participation throughout the study

This study is examining lived experiences of women with CKD and their decision making around pregnancy. The topic is complex and highly emotive. We anticipate that women who take part in this research will be particularly interested in the research outcomes and shared decision‐making tools. We will encourage active participation throughout by creating opportunities to share learning and for patients to feed into the research such as creating a patient reference group, invitation to attend data analysis meetings across, presentations at patient group meetings, and opportunities to co‐present research and co‐write academic articles.

### 3.9 Dissemination of research

We will work with our patient co‐applicant and stakeholders to produce public facing summaries of our findings, which will be distributed via social media, and the renal charities and national research centres and groups. This will include newsletters, plain English summaries of progress and findings, and an infographic summarising the main findings of the study. To facilitate adoption of patient‐centred care and shared decision making throughout the NHS, we will share our findings in other long‐term condition forums (e.g., organ transplant groups, rheumatology, diabetes), where similar complex decisions around starting families are faced by patients and their families.

An impact case study will be created on the Wales Kidney Research Unit webpage and we will present at their annual meeting. We will connect with Welsh renal charities and present findings at their meetings. We will present our work at the UKKW conference, NHS Research and Development conferences, and MDT team meetings to share our findings and demonstrate the benefits of multidisciplinary research and coproduction with patient groups.

Outputs will include

1. baseline demographic, clinical and psychosocial data from a cohort of women of reproductive age with kidney disease in Wales, with consent to follow‐up contact so that longitudinal data can be collected prospectively in future research;
2. theoretical model of the way in which women make decisions about pregnancy and family planning, by applying the TDF to the analysis of the survey and interview qualitative data;
3. at least two publications in peer‐reviewed journals (baseline survey data, shared decision‐making intervention development paper);
4. a lay summary, infographic and brief video clip for patients and members of the public.

#### Following up the work

Having identified needs and developed a model of shared decision making for women with CKD in relation to pregnancy, we will need to move to the next stage of development of the shared decision‐making intervention; developing a prototype and alpha testing. This study will provide the basis for future funding applications led by the co‐applicants (e.g., BRS/KCUK, the MRC Public Health Intervention Development scheme, NIHR) for pilot testing, refinement of the intervention, and subsequent rigorous evaluation of its implementation in UK clinical practice. We will also seek to build on the online survey cohort as the programme of work picks up momentum, and we will explore opportunities for data linkage with RaDaR and routinely collected data in systems such as Secure Anonymised Information Linkage (SAIL) databank.

## 4 DISCUSSION

This study was awarded funding in January 2020 and was set up during the COVID‐19 pandemic. We undertook an assessment of any impact to the study should movement continue to be restricted Many of the study processes and procedures were only designed for virtual sharing in the first instance, for example the survey is online only. The one to one interviews can be completed over the phone or via a web app and the preparation documents (FORM F) can be sent via the post—or virtually if the person to be interviewed feels comfortable completing the timelines on a home computer. The remainder of the processes including MDT engagement can be completed via the various online networks and forums which have been set up as a result of COVID‐19. Charities and partners have fast tracked several online communication networks and forums (e.g., Facebook and twitter chats and coffee morning) all of which will only benefit the recruitment and engagement of participants with this study. We have already found that due to social distancing many people living with kidney disease are seeking more active and meaningful activities they can participate in while shielding—which they may need to do (as kidney patients) depending on current guidance and individual clinical recommendations. We do not believe that future potential restrictions on movement will have any significant impact on the delivery of the study.

### 4.1 Limitations

The study primarily focuses on Welsh participants. Younger adults, for example 16–18 are excluded from this sample as are older participants over 51. BAME perspectives are likely to be low as Wales has a predominately white population. We are unable to include interviews and focus groups with professionals due to time and resource restrictions.

## CONFLICT OF INTEREST

No conflict of interest was declared by the authors in relation to the study itself. Note that Jane Noyes is a *JAN* editor but, in line with usual practice, this paper was subjected to double blind peer review and was edited by another editor.

### Peer Review

The peer review history for this article is available at https://publons.com/publon/10.1111/jan.14803.

## Supporting information

Data S1

Data S2
