# Supplementary material for: Engaging and supporting women with chronic kidney disease with pre‐conception decision‐making (including their experiences during COVID 19): A mixed‐methods study protocol
Source: J Adv Nurs. 2021 Mar 3;77(6):2887–97. doi: 10.1111/jan.14803 (PMC8014614; doi:10.1111/jan.14803)
Supplement: Supplementary file 1 — Data S1 [file JAN-77-2887-s001.pdf]

Researchers in Wales are working in collaboration with the NHS to learn more about women's experiences of kidney disease, decisions about having children, their experiences of pregnancy and having children.

Hello,

We want to make you aware of a new research study. If you are a woman living in the UK and aged between 18-50 you might be interested in sharing your thoughts and experiences with us.

We invite you to share your views about your decisions about having children whilst living with kidney disease, including your experiences over COVID 19 if relevant. This will help to better understand women's experiences and improve health and social care services.

The study is funded by the British Renal Society and Kidney Care UK.

This information has been given or sent to you on behalf of the study team. We would be really grateful if you could help by completing a short anonymous online survey that should take around 20-30 minutes. At the end of the survey is the option to share your contact details with the research team if you would be interested in taking part in an interview. Participation is voluntary and it is up to you to decide whether or not to take part. Even if you decide to take part, you are still free to withdraw, up to the point where you submit your survey responses.

If you have already received an invitation from another source, please pass this invitation onto another who you think might be interested in participating.

Please click on the link https://cardiff.onlinesurveys.ac.uk/ckd-engage or scan the QR code for further information and to take part.

Thank you for taking the time to read this email and we look forward to receiving your responses.

Yours sincerely,

Dr Leah Mc Laughlin
on behalf of the Pregnancy Choices with Kidney Disease research team
Wales Kidney Research Unit
l.mclaughlin@bangor.ac.uk
02921848469

Mae prifysgolion yng Nghymru yn gweithio ynghyd â'r NHS i ddysgu mwy am brofiadau menywod o glefyd yr arennau, penderfyniadau ynghylch cael plant, eu profiadau o feichiogrwydd a magu plant.

Helo,

Rydym am eich gwneud yn ymwybodol o astudiaeth ymchwil newydd. Os ydych chi'n fenyw sy'n byw yn y DU, ac rhwng 18-50 oed, efallai bydd gennych ddiddordeb mewn rhannu eich meddyliau a'ch profiadau gyda ni.

Rydym yn gwahodd menywod i rannu eu barn am benderfyniadau ynghylch cael plant wrth fyw gyda chlefyd yr arennau, gan gynnwys profiadau dros COVID 19 os yn berthnasol. Y gobaith yw bydd hyn yn helpu ni i ddeall profiadau menywod yn well, a gwella gwasanaethau iechyd a gofal cymdeithasol.

Ariennir yr astudiaeth gan Gymdeithas Arennol Prydain a Kidney Care UK.

Mae'r wybodaeth hon wedi iw hanfon atoch ar ran tîm yr astudiaeth. Byddem yn ddiolchgar iawn pe gallech helpu trwy gwblhau arolwg byr (di-enw) ar-lein, a ddylai gymryd tua 20-30 munud i'w gwblhau. Ar ddiwedd yr arolwg mae opsiwn i rannu'ch manylion cyswllt gyda'r tîm ymchwil pe bai gennych ddiddordeb mewn cymryd rhan mewn cyfweliad rhagor. Mae cyfranogiad yn wirfoddol, a chi sydd i benderfynu a ddylid cymryd rhan ai peidio. Hyd yn oed os penderfynwch cymeryd rhan, rydych yn dal yn rhydd i dynnu'n ôl (heb rhoi rheswm) ar unrhyw amser cyn cyflwyno eich ymatebi i'r arolwg

Os ydych eisoes wedi derbyn gwahoddiad gan ffynhonnell arall, croeso i chi ddrosglwyddo y gwahoddiad hwn i rhywun arall a all fod â diddordeb mewn cymeryd rhan.

Cliciwch ar y wefan: https://cardiff.onlinesurveys.ac.uk/ckd-engage neu sganiwch y cod QR am fwy o wybodaeth, neu i gymeryd rhan.

Diolch i chi am gymeryd yr amser i ddarllen yr e-bost hwn, ac edrychwn ymlaen at dderbyn eich ymatebion.

Yr eiddoch yn gywir,

Dr Leah Mc Laughlin
ar ran y tîm ymchwil Dewisiadau Beichiogrwydd gyda Clefyd yr Aren
Uned Ymchwil Arennau Cymru
l.mclaughlin@bangor.ac.uk
02921848469

Pregnancy choices with kidney disease

Participant Information Sheet

We are undertaking a new research study to learn more about how women make decisions about having or not having children whilst living with kidney disease. We are interested in women's views, attitudes and experiences of family planning and pregnancy (if applicable). We would like to invite you to take part in this study in order to help us learn.

Introduction

Who are we?

Our team includes people with different research expertise drawn from throughout Wales, including: The Wales Kidney Research Unit (WKRU, an All-Wales strategy for the study of diagnosis, prevention, treatment and social context of kidney disease, http://www.kidneyresearchunit.wales), Bangor University, Cardiff University, Cardiff Metropolitan University and Cardiff and Vale University Health Board. We have been funded by the British Renal Society and Kidney Care UK to undertake this important research. Cardiff and Vale University Health Board are sponsoring this study.

Background to this study

In this study we want to better understand the key factors that influence decision making from women's perspectives. The information we collect will help us design new tools for healthcare professionals to better engage with and support women with potentially complicated and emotional decisions about having children.

Why we are asking for your help?

We know very little about this important topic. You have been asked to take part in this study as a woman living with kidney disease, and are aged between 18-50. Sharing your views and opinions will help us learn and improve care and support for women living with kidney disease in future.

How can I help?

By completing a short online survey. We will also be inviting a small number of women to take part in an informal interview over the phone or online video call.

We would like to hear from all women with kidney disease aged 18-50, it does not matter if you have children or not. We would especially like to hear from women whose kidney disease has had a direct impact on their pregnancy experiences or decisions about having children.

Learning from the multiple perspectives is vital if we are to develop ways to support women through complex decisions and experiences in future. We would like to hear from women who are currently pregnant, have non-biologically related children, are considering adoption, same sex couples, single parents and women who are not in a relationship. If you have sadly had a miscarriage or stillbirth we would still very much like to hear from you, but only if the time is right for you. We would also like to hear from women who have been pregnant or given birth over the COVID 19 pandemic.

What does taking part involve?

Filling in an online survey:
You will be asked to take part in an anonymous online survey where you will be presented with a series of questions. These questions will ask about your; kidney disease, current circumstances, choices about pregnancy, health and wellbeing, communication with healthcare professionals, information needs, impacts of COVID 19, and your support networks.

The whole survey will take approximately 20-30 minutes. Once you have completed the survey, you will be taken to a page with information on organisations and resources that may be of help.

Informal interviews so that we can listen to your views and experiences in more detail:
We would like to listen carefully to some women's stories in more detail. At the end of the survey you will be asked if you would be interested in participating in a more detailed interview (approx.1 hour) with a member of the research team over the telephone, an online video call, or face to face meeting depending on which you prefer (face to face meetings will only be an option if and when it is safe to do so). If the answer to this is yes, you can enter your contact details and we may get in touch to arrange to talk at a time and in a way that suits you. With your permission, our conversation will be audio-recorded, but there will be no video recording.

You can also enter your details if you want to be kept updated about the study findings, news and other opportunities to take part in research on this topic. You do not have to complete this if you do not want and would rather remain anonymous.

What will happen to the information I give?
You can choose what you tell us. Our collaborators and partners have kindly agreed to send out an online link to this survey on our behalf. We do not know who has been emailed with this link and any healthcare professionals will not know who has replied to the survey.

The data you provide will be anonymised and analysed by members of the research team. Audio-recordings will be transcribed and anonymised by a transcription service employed by Bangor University, where a confidentiality agreement is in place. They will be stored on a secure password protected university hard drive for 5 years. Only members of the researchers team at Bangor University will have access to them. Anonymised quotes may be shared in research publications. It will not be possible to identify you from these quotes. Anonymised transcripts may be used for educational purposes such as an MSc or PhD student study

All identifiable data collected will be kept confidential and used for research purposes only. It will be stored in compliance with the Data Protection Act. We will not share any of your details unless you tell us something that makes us seriously concerned about your or another person's safety such as a child or vulnerable adult.

Cardiff and Vale University Health Board (C&VUHB) is the sponsor for this study based in the UK. We will be using information from your surveys and interviews in order to undertake this study and will act as the data controller for this study. This means that we are responsible for looking after your information and using it properly. Bangor University will keep identifiable information about you for 5 years after the study has finished until September 2026.

Your rights to access, change or move your information are limited, as we need to manage your information in specific ways in order for the research to be reliable and accurate. If you withdraw from the study, we will keep the information about you that we have already obtained. To safeguard your rights, we will use the minimum personally-identifiable information possible.

You can find out more about how we use your information at https://www.hra.nhs.uk/planning-and-improving-research/policies-standards-legislation/data-protection-and-information-governance/gdpr-guidance/ or by contacting the research team.

Bangor University will collect information for this research study in accordance with our instructions. Bangor University will keep your name and contact details confidential and will not pass this information to C&V UHB. Bangor University will use this information as needed to contact you about the research study, and make sure that relevant information about the study is recorded for your care, and to oversee the quality of the study. Certain individuals from C&V UHB and regulatory organisations may look at your medical and research records to check the accuracy of the research study. C&V UHB will only receive information without any identifying information. The people who analyse the information will not be able to identify you and will not be able to find out your name, or contact details.

What are the possible benefits of taking part?

We cannot promise that taking part will benefit you directly although many people welcome the opportunity to share their views and experiences. The information we gain will help us understand more about women who live kidney disease needs when planning to have children and to develop new shared decision making tools to improve their health and social care support in future.

What are the possible disadvantages and risks of taking part?

This is a low risk study nonetheless some of the questions ask you to think about topics which may be sensitive to you for example if you have had a complicated pregnancy, miscarriage or still birth. You may find this upsetting or distressing. You do not have to answer any question you do not want to. We have provided contact details and links to relevant support agencies at the end of the survey and at the bottom of this information leaflet.

What if I have concerns about this study?

If you have a concern about any aspect of this study, you should ask to speak to a member of the research team who will do their best to answer your questions. The Chief Investigator is Dr Sian Griffin, email sian.griffin2@wales.nhs.uk. You can also

contact us on any of the channels provided at the end of this sheet. If the research team are unable to resolve your query and/or you would like to speak to somebody outside of the research team you can contact the Cardiff and Vale Research manager on CAV_research.development@wales.nhs.uk.

What will happen to the results of the research study?
We will share the results of this study via newsletters, presentations, academic papers, reports and visual graphics with the multiple key stakeholders including; researchers, multi-disciplinary health and social care workers, policy makers, people living with kidney disease, charity partners, research participants and other key stakeholders. Findings will also support the development of new shared decision-making tools and future research into the field. It will not be possible to identify any individuals from reports or presentations.

Do I have to take part?
No, it is entirely up to you. Even if you agree to take part, you can opt out at any time. You do not have to give a reason for opting out. You can choose to skip any questions that you would prefer not to answer. You can opt out from the survey at any time by exiting the browser. Your responses will be anonymous, so we will be unable to withdraw your data once you have completed and submitted your questionnaire.

If you take part in an interview, you can skip any questions you would rather not answer or stop at any point if you do not want to continue. You will be able to ask that your interview, or specific sections of your interview, be withdrawn for 14 days after your interview by contacting the research team. After this point, your responses will be anonymised for inclusion in the analysis and it won't be possible to withdraw your data. Our contact details are below. We look forward to receiving your completed surveys and contact forms and learning from your opinions and experiences.

What's next if you would like to help?
Thank you. Please click on the following link which will take you to the consent FORM C and the online survey FORM D.

https://cardiff.onlinesurveys.ac.uk/ckd-engage

Further information and contact details

Please visit our website, Twitter and Facebook pages for project information, updates, and contact details for the research team.

Twitter: @Kidneypregnancy

Facebook: www.facebook.com/Pregnancy-choices-with-kidney-disease-106902844446942

Website: www.kidneyresearchunit.wales/impact-case-studies.htm?id=34

SURVEY DIRECT LINK: https://cardiff.onlinesurveys.ac.uk/ckd-engage

You can also contact the research team directly
Dr Leah Mc Laughlin (l.mclaughlin@bangor.ac.uk) (Tel. +44(0)2921848469)

This is study funded by the British Renal Society and Kidney Care UK.

Sources of support

If you have any concerns about your health and well-being, please talk to your GP or a member of your usual kidney care team. Here is a list of external organisations you may also find useful:

- Paul Popham Renal Fund, http://paulpophamfund.co.uk
- Kidney Care UK, https://kidneycareuk.org
- Kidney Wales, https://www.kidneywales.cymru
- Relate: Relate specialises in relationship support throughout the UK. https://www.relate.org.uk
- Mind: Mind campaigns to improve services, raise awareness and promote understanding' of mental health issues. http://www.mind.org.uk/
- British Infertility Counselling Association (BICA). BICA aims to aid those with fertility problems at any stage of their life. http://bica.net/
- SANDS: https://www.sands.org.uk/ for support with stillbirth & neonatal death

pregnancy choices with kidney disease

Page 1: Information about the study

Would you like to complete this survey in Welsh ☐ or English ☐ link opens to Welsh or English version.

Please click on link below to read the information sheet before you decide whether you would like to take part in this survey.

https://static.onlinesurveys.ac.uk/media/account/88/survey/622453/question/FORM_B_Participant_Information_jsyo3tr.docx

If you have any questions, please contact the research team at the Wales Kidney Research Unit. Tel: 02921848469. Email: l.mclaughlin@bangor.ac.uk

Thank you for your time.

1. I confirm that I have read and understand the Participant Information Sheet dated (………) for the above study and have had the opportunity to ask questions.

   (Please note that if you have any questions, you can contact the research team on ……………)

   a. Yes
   b. No

2. I understand that my participation is voluntary and that I am free to withdraw at any time without giving any reason. I do not have to answer any questions that I don't want to answer.

   a. Yes
   b. No

3. I agree to take part in the Pregnancy Choices with Kidney Disease online survey

   a. Yes
   b. No

(If participants answer NO in response to any of those questions, they will be redirected to the following message:

Thank you for your interest in this survey. We are currently only able to invite people to take part in the survey who have agreed to the above statements.
Please visit our website, Twitter and Facebook pages for project information, updates and contact details for the research team.
Twitter: @Kidneypregnancy
Facebook: www.facebook.com/Pregnancy-choices-with-kidney-disease-106902844446942
Website: www.kidneyresearchunit.wales/impact-case-studies.htm?id=34

ELIGIBILITY SCREENING QUESTIONS

1. Have you been diagnosed with Kidney Disease by a doctor? (Please select Yes or No)
   a. Yes
   b. No
2. Do you currently live in the UK (Please select Yes or No)
   a. Yes
   b. No
3. Are you aged between 18 and 50 years? (Please select Yes or No)
   a. Yes
   b. No
4. Are you female
   a. Yes
   b. No

[If participants answer NO in response to any of these questions they will be redirected to the following message:

Thank you for your interest in this survey. We are currently only able to invite people who have been diagnosed by a doctor with Kidney Disease, are resident in the UK, are aged between 18 and 50 years and female in this survey. We hope to build on this work in the future, so please visit our website, Twitter and Facebook pages for project information, updates and contact details for the research team.

Twitter: @Kidneypregnancy
Facebook: www.facebook.com/Pregnancy-choices-with-kidney-disease-106902844446942
Website: www.kidneyresearchunit.wales/impact-case-studies.htm?id=34

Welcome to the pregnancy choices with kidney disease survey. The main focus of this survey is experiences of decisions about pregnancy and having children but COVID 19 has had an impact on all of our day to day lives. We will therefore also ask about your views and experiences for care before, during and after any impacts of COVID 19.

1) **MY KIDNEY DISEASE QUESTIONS**

a) **What is the cause of your kidney disease?**
   i) **Polycystic Kidney Disease**
   ii) **Diabetes**
   iii) **Reflux nephropathy**
   iv) **Nephrotic syndrome**
   v) **Lupus**
   vi) **IgA nephropathy**
   vii) **Vasculitis**
   viii) **Another type of glomerulonephritis**
   ix) **I'm not sure**
   x) **Other** (please state**)** ___________________________

b) **What is your Chronic Kidney Disease (CKD) stage?**

CKD stage 1 ☐CKD stage 2 ☐ CKD stage 3 ☐CKD stage 4 ☐ CKD stage 5 ☐ I'm not sure ☐I am on dialysis ☐ I have a transplant ☐

i) If 'I have a transplant ticked', Who donated your current transplant? (tick one)
   (a) You received a transplant directly from a relative or friend
   (b) You received a transplant via the donor sharing scheme (your relative or friend donated to the donor sharing scheme)
   (c) You received a transplant from a living donor you did not know ("altruistic donor")
   (d) You received a deceased donor transplant

(2) How long have you had your transplant? _ years or less than a year ☐
(3) Is this your first transplant? Yes ☐ No ☐ (if no)
   (a) How many transplants have you had? _
(4) Is your transplant working well? Yes ☐ No ☐

(5) Did you receive your transplant before needing dialysis? Yes ☐ No ☐ (If no)
   (i) What type of dialysis did you have before your transplant? Exchanging fluids with bags while you sleep, Automated Peritoneal Dialysis (APD) ☐ Exchanging fluids with bags 4 times a day, Continuous Ambulatory Peritoneal Dialysis (CAPD) ☐ Home Haemodialysis Daytime ☐ Home Haemodialysis overnight, also called 'nocturnal dialysis' ☐ Dialysis in a hospital or stand-alone Unit ☐
   (ii) How long were you on dialysis? _ years or less than a year ☐

ii) If 'I am on dialysis' ticked additional questions open. Which dialysis treatment are you CURRENTLY on?
(1) Exchanging fluids with bags while you sleep, Automated Peritoneal Dialysis (APD) (if yes, additional questions open)
   (a) how long have you been on this treatment? _ years or less than a year ☐

(2) Exchanging fluids with bags 4 times during the day, Continuous Ambulatory Peritoneal Dialysis (CAPD) (if yes, additional questions open)
   (a) how long have you been on this treatment? _ years or less than a year ☐
   (b) Does a nurse specialist come and assist you with your dialysis sometimes called 'Assisted CAPD' Yes ☐ No ☐

(3) Home Haemodialysis overnight, 'nocturnal dialysis' (if yes, additional questions)
   (a) how long have you been on this treatment? _ years or less than a year ☐

(4) Daytime Home haemodialysis 'daytime' (if yes, additional questions)
   (a) how long have you been on this treatment? _ years or less than a year ☐

(5) Hospital or Unit based haemodialysis (if yes, additional questions)
   (a) how long have you been on this treatment? _ years or less than a year ☐

iii) Are you currently on the transplant waiting list Yes ☐ No ☐

c) **In general, would you say your health is:** Excellent ☐ Very good ☐ Good ☐ Fair ☐Poor

2) YOUR CHOICES AND CURRENT CIRCUMSTANCES

In this section, we would like to ask you some questions about your decisions about having children, or having more children and what stage you are in now. These are very personal topics and we would like to understand what is most important to women, whether they would like additional information or support and from whom, when they are making decisions about whether or not to have children.

1. Do you have any children already? Please tick one option.
   a. Yes
   b. No

If yes, go to Q 2. If no, go to Q 10

2. How many children do you have? ____
3. Please tell us your children's ages in years. ____
4. How many of your children (if any) are your biological children? That is, they are genetically related to you. ____
5. If you have biologically related children, how were they conceived?
   a. Naturally
   b. With fertility treatment (e.g. IVF)
   c. Would rather not say
   d. Not applicable

6. If you have children that are not biologically related to you, can you please tell us if they are (Tick all that apply):
   a. Adopted
   b. Stepchildren
   c. Children through a surrogate
   d. Foster children
   e. Not applicable

7. Do you think becoming a parent has had an impact on your physical health?
   a. Yes – my health got **better**
   b. Yes – my health got **worse**
   c. No – my health is about the same as it was before the pregnancy

d. I'm not sure

8. Do you think that becoming a parent has had an impact on your mental health?
   a. Yes – my health got **better**
   b. Yes – my health got **worse**
   e. No – my health is about the same as it was before the pregnancy
   c. I'm not sure

9. Please tell us a bit more about any impact you think becoming a parent has had on your health and well-being.

____

10. Would you like to have children in the future (or have more children if you have children already)?
    a. No, I do not want to have any children (or any more children)
    b. I am currently pregnant
    c. Yes, I would like to become pregnant
    d. Yes, I am receiving fertility treatment/planning on having fertility treatment
    e. Yes, I would like to have a child but don't plan on getting pregnant (adoption/surrogacy)
    f. I'm not sure because other…. , please specify____________________

11. What were the main things that influenced your decision about whether or not you would like to have children (or more children)? These could include conversations with health and social care professionals, lifestyle, family or social influences, childhood influences or anything else.

12. Has the COVID 19 pandemic had any influence on your decisions about **becoming pregnant or having (more) children**. Yes ☐ No ☐ Please give further details (e.g. changes to your usual care from your renal team, delayed transplant, risk of infection, employment, welfare and/or social support).

3) PREGNANCY, HEALTH AND WELL-BEING

We would like to find out more about women with Kidney Disease, who have been pregnant, feel that their pregnancy had an impact on their health and well-being.
If you have not been pregnant, please tick 'no' below and you will be taken to the next section of the survey.

a) Have you ever been pregnant?
   i) Yes (goes to Q.b)
   ii) No (goes to section 4).
b) If yes, please tell us how many times you have been pregnant__________________________________
c) Of these how many would you say were 'planned' ___ and how many were 'unplanned' ___
d) Have you been pregnant or given birth during the COVID 19 pandemic (23rd March to present)?
e) Thinking about your most recent pregnancy, did your pregnancy have an impact on your physical health?
   i) Yes- my health got **better**
   ii) Yes – my health got **worse**
   iii) No – my health is about the same as it was before the pregnancy
   iv) I'm not sure
f) Thinking about your most recent pregnancy, did your pregnancy have an impact on your mental health?

i) Yes- my mental health got **better**
ii) Yes – my mental health got **worse**
iii) No – my mental health is about the same as it was before the pregnancy
iv) I'm not sure

g) Please tell us a bit more about any impact you think your pregnancy or pregnancies have had on your health and well-being.
____

h) Have you experienced any of the following? That is, miscarriage, termination of pregnancy, or a still birth. Please select all that apply.
i) Abortion
ii) Miscarriage
iii) Ectopic pregnancy
iv) Still birth
v) None of the above
vi) Would rather not say
vii) If you would like to tell us more about your experience of pregnancy, please use the space below. For example, were you offered enough support, if you had a partner were they offered support, what information would you have liked (if any)?_______________________________________

4) **COMMUNICATION WITH HEALTH AND SOCIAL CARE PROFESSIONALS**

In this section, we will be asking you some questions about communication between yourself and your health and social care team. This includes your Kidney Care team, GP, and obstetrics team (if relevant).

a) Ideally, how involved would you like to be in decisions about the management of your disease?
i) I prefer to leave all decisions regarding treatment to my doctor
ii) I prefer that my doctor makes the final decision about which treatment will be used, but seriously considers my opinion
iii) I prefer that my doctor and I share the responsibility for deciding which treatment is best for me
iv) I prefer to make the final decision about my treatment after seriously considering my doctor's opinion
v) I prefer to make the decision about which treatment I receive

b) Have any healthcare professionals discussed your preferences for having children with you? (Please tick yes or no)
i) Yes
ii) No
(1) If yes, who spoke to you about this? (e.g. your family doctor, your kidney care team, or a family planning clinic)____
(2) How much effort was made to help you understand your options about having children and managing your conditions? No effort was made 0 1 2 3 4 5 6 7 8 9 Every effort was made
(3) How much effort was made to listen to the things that matter most to you? No effort was made 0 1 2 3 4 5 6 7 8 9 Every effort was made
(4) How much effort was made to include what matters most to you in choosing what to do next? No effort was made 0 1 2 3 4 5 6 7 8 9 Every effort was made

c) Have your kidney health and social care team considered whether or not you would like to have children when talking about your treatment options (e.g. types of medication, dialysis, transplant)? Please circle a number from 0 (not considered at all) to 4 (fully considered):
Not considered at all 0 1 2 3 4 Fully considered

d) Are you taking any medications to manage your kidney disease (e.g. high blood pressure tablets, immunosuppressants)? Yes ☐ No☐ If yes, please list these here:……………………….
e) Did your doctor stop any medications when you started family planning or when you became pregnant? Yes ☐ No☐ N/A ☐ I'm not sure ☐ If yes, please list these here:……………………….
   i) Would you like to tell us anything further about the impact of changes to your medication (e.g. were you worried or concerned, was your health affected, did you understand why there were changes, did your doctor fully explain any changes etc.)
   ____

f) Would you like to have a conversation with your healthcare professionals about decisions regarding having children?
   i) Yes
   ii) No
      (1) If Yes: **Who** would you like to speak with about making your decision about whether or not to have children, e.g. your kidney care doctor, GP, a counsellor, etc?
         (a) kidney doctor
         (b) kidney specialist nurse
         (c) G.P
         (d) counsellor/psychologist
         (e) social worker
         (f) other…………………….
      (2) **Who** would you like to bring up the topic of having children?
         (a) The healthcare professional
         (b) You
         (c) Other………………………………….
      (3) **When** do you think it is most useful for women to speak to a healthcare professional about whether or not they would like to have children (e.g. at what age, when making treatment decisions, or only when women are starting to think about having a family)?____

g) Listed below are some things involved in making choices about having children. This can include whether to have children, or to have another child. Please show how confident you feel in doing these things by selecting a number from 0 (not at all confident) to 4 (very confident). I feel confident that I can:

a.	Find information about the benefits of each choice	Not at all confident	0	1	2	3	4	Very confident
b.	Find information about the risks of each choice	Not at all confident	0	1	2	3	4	Very confident
c.	Understand information enough to be able to make a choice	Not at all confident	0	1	2	3	4	Very confident
d.	Ask questions without feeling dumb	Not at all confident	0	1	2	3	4	Very confident
e.	Ask for advice from healthcare professional about the best option for me	Not at all confident	0	1	2	3	4	Very confident
f.	Figure out the choice that best suits me	Not at all confident	0	1	2	3	4	Very confident
g.	Let the clinic team know what's best for me	Not at all confident	0	1	2	3	4	Very confident

h) From your experiences, has the COVID 19 pandemic influenced the *quality of* communication with your healthcare professionals? Yes ☐ No ☐ N/A☐ Would you like to tell us some more details e.g. virtual appointments, online applications, understanding advice etc? ___________________________

i) Is there anything else that you would like to tell us about your experiences of communicating with health professionals about your plans for having, or not having, children?____

5) CONTRACEPTION/BIRTH CONTROL

a) Have any healthcare professionals discussed options for contraception with you? That is, a method to stop you from becoming pregnant, also known as 'birth control' or 'protection'? (Please select yes or no)
   i) Yes
   ii) No If **yes**, who spoke to you about this? (e.g. your GP, specialist doctor or nurse, a family planning clinic)_______________________________________

b) Do you feel that you have enough information about your contraception options?
   i) Yes
   ii) No
   iii) Not sure If you answered **no** or **not sure**, please tell us what information would you like to help you decide on your birth control options?____

c) Are you currently using any birth control (also known as contraception or protection)? Please tick all that apply.
   i) I'm not using any birth control
   ii) Abstinence (not having sexual intercourse)
   iii) Condoms
   iv) Combined pill (combined oestrogen and progesterone pill)
   v) Mini pill (progesterone only pill)
   vi) Progesterone implant or injection (Depo)
   vii) An intrauterine device (IUS or IUD) (also known as a hormone coil, e.g. Mirena, Skyla, Lyetta, copper coil or a copper IUD)
   viii) Implant (Nexplanon)
   ix) Diaphragm, cap or ring
   x) Fertility awareness (also known as the rhythm method, calendar method or natural cycles)
   xi) Withdrawal method (when your partner withdraws before he reaches orgasm/ejaculates)
   xii) My partner has had a vasectomy
   xiii) Other (please specify)_________________________

d) What were the main things that influenced your choice of contraception? _________________

6) INFORMATION NEEDS

a) Do you feel that you have enough information from your healthcare professionals to help you decide whether or not you would like to have children? (please tick one option)
   i) Yes
   ii) No
   iii) Not sure

If you would like to have children or haven't decided yet, please continue to Question B. If you have decided not to have children, please go to Section 7.

b) Please score from 0 to 4 how important it is for you to have more information for each statement

	Not at all important (0)	A little (1)	Somewhat important (2)	Very important (3)	Extremely Important (4)
a. Managing impact of my illness on sex and relationships					
b. If my illness might affect my ability to become pregnant					
c. Fertility testing options					
d. Fertility treatment options (including IVF)					
e. Fertility preservation (freezing eggs so that they can be used at a later time)					
f. Risks of passing on my kidney disease					
g. Other options for starting a family (e.g. adoption, fostering or surrogacy)					
h. What I would need to do to prepare for a pregnancy (e.g. changing medications, talking to my healthcare team)					
i. If my condition increases my risk of miscarriage or still birth					
j. My options for giving birth					
k. Whether I would be able to breastfeed					

c) **If you would like to have extra information how would you like to receive it? Tick all that apply.**

a. Face-to-face conversation with your kidney consultant/ specialist nurse
b. Face-to-face conversation with your gynaecologist/obstetrics specialist
c. Online conversation with your kidney consultant / specialist nurse
d. Online conversation with your gynaecologist/obstetrics specialist
e. Telephone conversation with your kidney consultant / specialist nurse
f. Telephone conversation with you gynaecologist/obstetrics specialist
g. A leaflet sent to you ahead of an upcoming kidney appointment
h. A leaflet given to you during your kidney appointment
i. A website
j. A video/DVD
k. An app (e.g. Patient View)
l. Peer to peer support
m. Other…………………………….

7) YOUR SUPPORT NETWORKS

a) The following questions ask about your support network.

	None of the time (1)	A little of the time (2)	Some of the time (3)	Most of the time (4)	All of the time (5)
a) Is there someone available to you whom you can count on to listen to you when you need to talk?					
b) Is there someone available to give you good advice about a problem?					
c) Is there someone available to you who shows you love and affection?					
d) Is there someone available to you to help you with daily chores?					
e) Can you count on anyone to provide you with emotional support (talking over problems or helping you make a difficult decision)?					
f) Do you have as much contact as you would like with someone you feel close to, someone in whom you can trust and confide?					
g) Has COVID 19 had an impact on your contact with people you feel close to and that you can trust and confide in?					
h) Are you currently married or living with a partner?	NO ☐		Yes ☐		

b) Would you like to tell us anything else about the impact of COVID 19 on your support networks?
____

c) Is there any advice that you would give to women with kidney disease who are in the process of deciding whether or not they would like to have children? ______________________________

d) Before this survey had you heard of the National Registry of Rare Kidney Diseases (RaDaR)? www.rarerenal.org Yes ☐ No. ☐ (*Pregnancy and CKD is a group in the renal registry, please speak to your kidney health care professional if you want to know more)*

8) **ABOUT YOU: GENERAL INFORMATION** The following questions ask for some general information about you. This is so that we can see whether we're capturing the views of a range of women from various backgrounds and from different countries

a) What is your age? _______________years

b) What is your current relationship status? Please tick one option.

i) Single

ii) Married or in a civil partnership

iii) Living with a partner

iv) In a long-term relationship

v) Widowed, divorced or separated

- **vi)** Rather not say

c) Do you have any of the following qualifications? (Please tick all that apply)
- **i)** Usual high school qualifications in your country at age 16
- **ii)** Usual high school qualifications in your country at age 18
- **iii)** A college or university diploma or degree
- **iv)** A higher degree or professional qualification (e.g. a Doctorate or Masters level degree)
- **v)** None of these qualifications
- **vi)** Other
- **vii)** Rather not say

d) How would you describe your current employment status? (Please tick all that apply)
- **i)** In full-time paid work, as an employee or self-employed
- **ii)** In part-time paid work, as an employee or self-employed
- **iii)** Unemployed and seeking work
- **iv)** Not employed and not currently seeking work
- **v)** In full-time education or training
- **vi)** In part-time education or training
- **vii)** Rather not say

9) Where do you live in the UK?

a) England ☐ Scotland ☐ Northern Ireland ☐ Wales ☐

i) If Wales, where in Wales? Isle of Anglesey ☐ Conwy ☐ Gwynedd Denbighshire ☐ Flintshire ☐ Wrexham ☐ Powys ☐ Ceredigion ☐ Pembrokeshire ☐ Carmarthenshire ☐Swansea ☐ Neath Port Talbot ☐ Bridgend ☐ Rhondda Cynon Taff ☐ Merthyr Tydfil Blaenau ☐Torfaen ☐ Monmouthshire ☐ Caerphilly ☐ Newport ☐ Cardiff ☐Vale of Glamorgan ☐

b) Which type of area do you live in?
- **i)** City
- **ii)** Large town
- **iii)** Small town
- **iv)** Village
- **v)** Hamlet
- **vi)** Isolated Dwelling

c) Please choose one option that best describes your race or ethnic background.
- **i)** White
- **ii)** Black/African/Caribbean
- **iii)** Hispanic
- **iv)** Asian
- **v)** Mixed/Multiple groups
- **vi)** Other
- **vii)** Rather not say

d) Please choose one option that best describes your gender?
- **i)** Female
- **ii)** Non-binary
- **iii)** Other……………….
- **iv)** Rather not say

e) Please choose one option that best describes your sexual orientation?
- **i)** Heterosexual or Straight
- **ii)** Gay or Lesbian
- **iii)** Bisexual
- **iv)** Other……….
- **v)** Rather not say

Thank you

Thank you for completing this questionnaire. If you have any concerns about your health and well-being, please talk to your GP or a member of your usual kidney care team. A list of other sources of support with issues covered in this survey is provided on the next page.

What happens next?
We will be carrying out in-depth interviews with a small number of women to find out more about their experiences. If you would be interested in taking part in an interview, please enter your contact details below:
Name:________________________
E-mail address:_________________
Phone number: _________________

Using the information you have given us, we will be looking at what information, tools to support decisions, and other forms of support women would like to help them with their decisions about having (more) children. If you would like to be updated with our findings and news about other opportunities to take part in research on this topic, please enter your e-mail address below: ___________
____

Please note: *Your contact details will be kept confidential, and will be held securely as set out in the UK Data Protection Act and EU General Data Protection Regulation. This information will only be accessible to the research team and we will only use your contact details for the purposes stated above. Your contact details will not be used for marketing and will not be shared with any third parties.*

Please visit our website, Twitter and Facebook pages for project information, updates, and contact details for the research team.

Twitter: @Kidneypregnancy
Facebook: www.facebook.com/Pregnancy-choices-with-kidney-disease-106902844446942
Website: www.kidneyresearchunit.wales/impact-case-studies.htm?id=34

If you have any questions or concerns about this project, please contact:

Dr Leah Mc Laughlin
Wales Kidney Research Unit
Bangor University
Heath Hospital
Cardiff CF14 4XN
E-mail: l.mclaughlin@bangor.ac.uk
Phone: 02921848469

Sources of support

If you have any concerns about your health and well-being, please talk to your GP or a member of your usual kidney care team. Here is a list of external organisations you may also find useful:

- Paul Popham Renal Fund, http://paulpophamfund.co.uk
- Kidney Care UK, https://kidneycareuk.org
- Kidney Wales, https://www.kidneywales.cymru
- Relate: Relate specialises in relationship support throughout the UK. https://www.relate.org.uk
- Mind: Mind campaigns to improve services, raise awareness and promote understanding' of mental health issues. http://www.mind.org.uk/
- British Infertility Counselling Association (BICA). BICA aims to aid those with fertility problems at any stage of their life. http://bica.net/

- SANDS: https://www.sands.org.uk/ for support with stillbirth & neonatal death

Pregnancy Choices with Kidney Disease

Consent form to interview

Please INITIAL each box

**I confirm that I have read and understood the participant information sheet Version 3 dated 05.06.20 I have had opportunity to consider the information and ask questions.**	
**I have had my questions answered satisfactorily.**	
**I understand that my participation is voluntary and that I can withdraw at anytime without giving a reason.**	
**I agree to being interviewed.**	
**I am happy for the interview to be recorded with an audio recording device which will be transcribed and anonymised.**	
**I understand that you will write a report about the study findings.**	
**I am happy for quotes from my interview to be included in the report and that my identity will not be revealed.**	
**I understand that information about me (including names and address) will be held by the research team and that this information will be kept strictly confidential.**	
**I agree to allow the information that I give to be used for educational purposes such as future MSc of PhD student research**	

______________________	______________________	______________________
Your name	Date	Signature
______________________	______________________	______________________
Name of researcher	Date	Signature of researcher

1 copy for participant, 1 copy for researcher

My interview: What to expect

Before the interview we would like you to understand what we would like to talk about, and for you to think about these issues. The interviews are very informal. We would like you to tell us about your experiences in your own words. You can chose what you would like to talk to us about. Depending on your own personal situation, this might include things like:

- Deciding to have or not to have children when you have kidney disease
- Unplanned pregnancy when you have kidney disease
- What it is like to plan a pregnancy when you have kidney disease
- Your experiences with healthcare professionals (good and bad)
- Any care and support that you have received outside of healthcare (e.g. charities, social services, partner, family, friends)
- Starting dialysis/having a transplant
- Difficulty with getting pregnant, or losing a baby
- How your illness affected you during pregnancy
- How your illness affected you after your baby was born
- How things are for you now
- Your experiences of pregnancy/giving birth over the COVID 19 pandemic
- Your experiences of care and support before, during and after the COVID 19 pandemic
- What you think about the future

You do not have to talk about anything that you don't want to. If there is anything you don't want to talk about, please let the researcher know before the interview or at any point during the interview. You are free to stop and end the interview at any point, without giving a reason.

Using the timeline

In some of our previous studies, participants have found it helpful to write some notes in advance, and then to talk through these notes with the researcher. We have enclosed a resource pack with some stationary and worksheets that you can use if you'd like to. **If you would prefer not to write anything down, that's also fine.**
Some women find a timeline useful for noting down some of the important things they'd like to tell us about. We have included a timeline template that you can fill in, which includes some blank boxes. You can write as little or as much as you like. You can also chose to create your own timeline without using the template.
You can use coloured pens or stickers to represent how you were feeling at certain points. You could also focus on how your kidney disease affected you at different times. If you would like to do this, you could use this key:

- Pain-red
- Tired–orange
- Mobility problems–blue
- Feeling sick or nausea–yellow
- Medication–green
- Healthcare support–purple

- Informal support from family and friends-pink

If you wish to use any other colours to represent different feelings, experiences and emotions, feel free to use colouring pens or pencils, but please include a key at the top. If you have any questions about preparing for your interview, please do not hesitate to contact us.

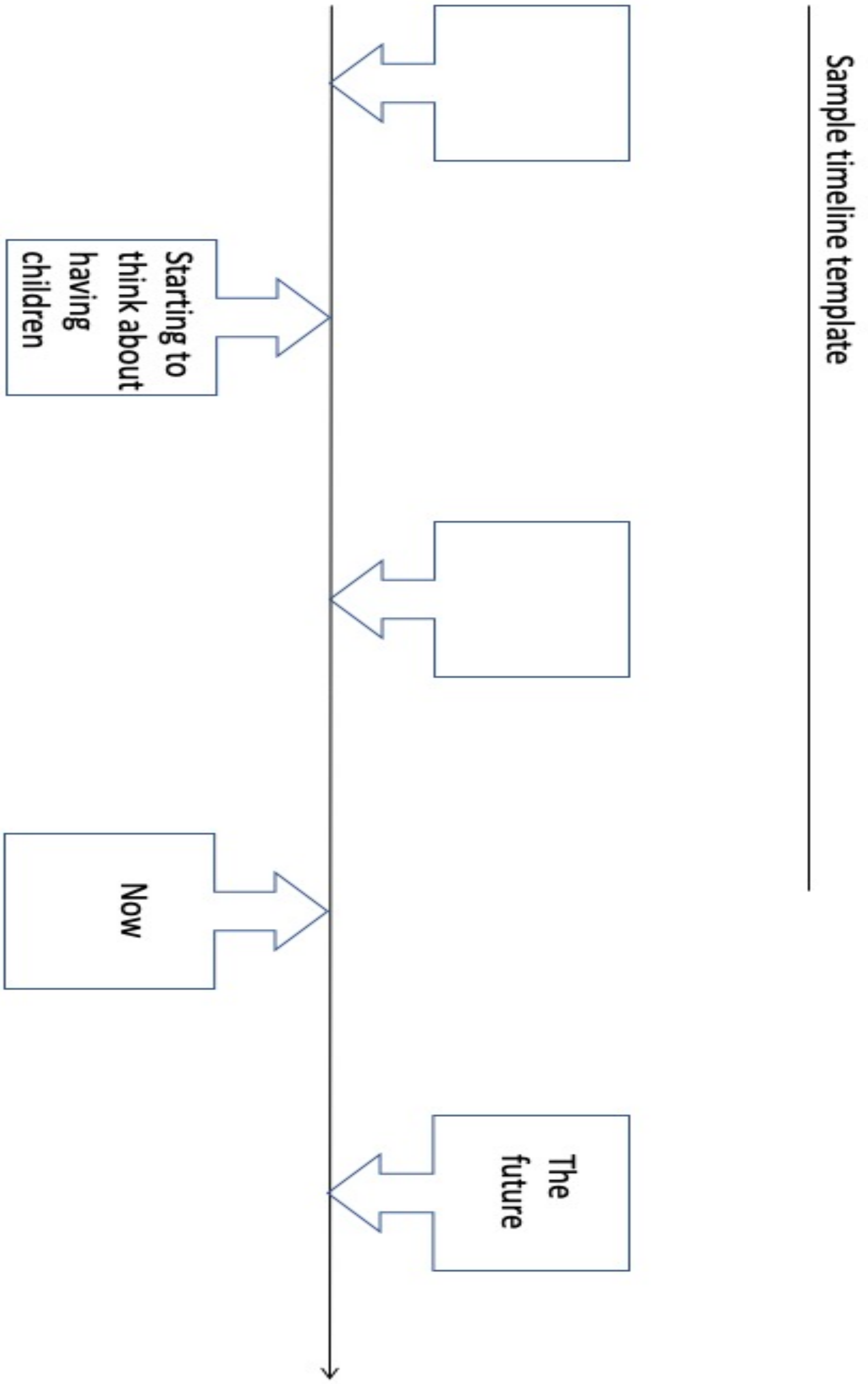

We are undertaking a study to learn more about women's experiences of kidney disease, decisions about having children and their experiences of pregnancy and having children.

pregnancy choices with kidney disease

Findings will help us develop new shared decision making tools to help better support women with the sometimes complicated and emotional decisions they may need to make about having children.

To hear more about this study, check if you are eligible to **take part and share your views you can get in touch** via any of the contact details below. You can also go directly to the **online survey via the link below or scan the QR code.** We look forward to hearing from you.

Survey link: https://cardiff.onlinesurveys.ac.uk/ckd-engage
Website: http://www.kidneyresearchunit.wales/impact-case-studies.htm?id=34
Email: l.mclaughlin@bangor.ac.uk
Tel: 02921848469
Twitter: @kidneypregnancy
