# Supplementary material for: Engaging and supporting women with chronic kidney disease with pre‐conception decision‐making (including their experiences during COVID 19): A mixed‐methods study protocol
Source: J Adv Nurs. 2021 Mar 3;77(6):2887–97. doi: 10.1111/jan.14803 (PMC8014614; doi:10.1111/jan.14803)
Supplement: Supplementary file 2 — Data S2 [file JAN-77-2887-s002.pdf]

Supplemental file 2. Ethical considerations, practical strategies and distress protocol
Version 1 01.05.20

Ethical considerations	Practical strategies
**Participant identification and recruitment**	
Access, Confidentiality, Regard	Work closely with the Multi-Disciplinary Teams to alert potential participants of the opportunity to take part in the study. Identify additional key stakeholders (charities and third sector organisations) to advertise the study, work with research networks and social media outlets (HealthWise Wales and suitable online forms to promote the study. Develop one way data flow to ensure appropriate sharing of data between organisations.<br><br>Make it clear that not all participants will be contacted for follow up interview. Ensure potential participants remain informed of study progress via social media and online newsletters. Signpost to RaDaR for women to be alerted to additional research and opportunities to take part.
Respect, Relevance	Ensure study materials are clear and sensitive to the many individual circumstances of potential participants. Advertise clearly that it is the womens individuals perspective that is needed and that this will be used to inform future research designs.
Compassion	Develop study materials that are sensitive, personable and accessible.
Informed choice, Support	Provide clear written and web based information about the study and researchers. Make it clear that it is entirely voluntarily to take part throughout. Provide timely responses to any potential questions or queries.
**The research survey**	
Informed consent	Provide an overview of the study and present opportunity for participants to access further information online. Obtain written consent before participants have option to proceed to the survey.
Support	Provide details of follow up services and signposting at the beginning and end of the survey. Simply and clearly introduce each section, use lay language throughout.
Confidentiality, Anonymity	Ensure option to share contact details is voluntarily and explain what and how we will use this data. Ensure surveys are securely stored and analysed anonymously.
**The research interview**	
Choice, respect	Agree convenient date, time, venue for research interview. Be mindful of dates that might coincide with any significant family events or anniversaries
Safety	Implement lone worker policy for researchers working alone in advance of the interviews
Safety, support	Researcher with experience of conducting sensitive interviews face to face, via telephone and online.
Choice, privacy	Provide the option of an interview face to face (where possible) or remotely, for example, via telephone.
Informed consent	Provide an overview of the study and present opportunity for participants to ask questions. Explain how the interview will proceed. Obtain written agreement to audio-record the interview and to use anonymous quotes in any presentation of the research. Provide participants with a copy of the signed consent form to keep.
Support	Discuss and agree avenues of post-interview support. Offer a follow up telephone call, and offer for the participant to get in touch with the research team if they wish to share anything further while the study is still open. Sign post to additional support services. Observe/listen for signs of distress during the interview. Discuss the option of pausing the recording or stopping the interview. Plan a natural break for refreshments. Provide timelines for women to tell their story in a way that suits them to facilitate memory and recall of potentially complex and sensitive times.

Supplemental file 2. Ethical considerations, practical strategies and distress protocol
Version 1 01.05.20

Confidentiality, anonymity	Ensure audio-recordings and transcripts are securely stored and electronic data are password protected. Assign a study code at the point of transcription.
**Post-interview processes**	
Support	If participant wants follow up call, arrange a convenient time to telephone the participant (normally in 24–48 h) to check on any issues the interview may have raised and to answer any questions. Redirect information about local support organisations. Offer this to participants if they consider it helpful and/or direct them to appropriate professionals to discuss any issues of concern. Establish if participants wish their general practitioner (GP) to be informed about their participation in the study and obtain written consent to proceed. Provide GP with information about the study at the time of notification.
Appreciation	Send participants a personal thank-you letter alongside a lay summary of findings offer an executive summary of the research findings.
Involvement	Provide participants with an opportunity to evaluate their experience of participating in research and sign post to RaDaR for further opportunities to participate.
Researcher Support	Determine support for the researcher from an individual with whom they feel comfortable and who is suitably qualified to provide support. Plan a debriefing session after each interview encounter. Utilise reflexive notes to guide the discussion.

Distress Protocol

During instances of participants becoming distressed during the interview process, the subsequent protocol will be followed.

Identifying Distress

The interviewer will be mindful of signs of distress in the participants throughout the interviews. Signs of distress to look out for will include:

• Exhibition of behaviours that indicate that the discussion has become too upsetting for them, including crying and an inability to continue for example.
• The participant verbally communicating that they are experiencing distress during the interview.

Response Stage 1

- The interview will be stopped.
- The participant will be offered a break, have a drink of water/ tea etc.
- The participant will then be asked if they would like to continue the interview or if they would prefer to discontinue. Should they wish to go on, the interview will resume.

Response Stage 2

If the participant elects to discontinue the interview,

- The interview will not continue.
- The interviewer will signpost where support can be obtained such as from their kidney care team, or charity support services or local support groups.
- The participants will also be reminded again of the contact details for SANDs, an organisation which provides support for stillbirths and neonatal deaths.

Response Stage 3

• At a later date, the interviewer will follow up with the participant with a courtesy call (with the participant's consent). If the participant feels strongly that they would still like to have their views and experiences heard – the interviewer will go through options (wait a while before rearranging, explore other methods rather than face to face etc).
